# Risk Factors for Aggressive Recurrent Respiratory Papillomatosis in Adults and Juveniles

**DOI:** 10.1371/journal.pone.0113584

**Published:** 2014-11-24

**Authors:** Turid Omland, Harriet Akre, Kathrine A. Lie, Peter Jebsen, Leiv Sandvik, Kjell Brøndbo

**Affiliations:** 1 Department of Otorhinolaryngology/Head and Neck Surgery, Oslo University Hospital, Oslo, Norway; 2 Department of Pathology, Oslo University Hospital, Oslo, Norway; 3 Department of Biostatistics, Oslo University Hospital, Oslo, Norway; 4 University of Oslo, Institute of Clinical Medicine, Oslo, Norway; 5 University of Oslo, Faculty of Dentistry, Oslo, Norway; University of Nebraska-Lincoln, United States of America

## Abstract

In this cohort study we examined whether gender, age at onset, observation time or human papillomavirus (HPV) genotype are risk factors for an aggressive clinical course in Recurrent Respiratory Papillomatosis (RRP). Clinical data from patient records comprised gender, age at onset, date of first endolaryngeal procedure with biopsy, date of last follow-up, total number of endolaryngeal procedures, and complications during the observation period. Disease was defined as juvenile (JoRRP) or adult onset (AoRRP) according to whether the disease was acquired before or after the age of 18. Aggressive disease was defined as distal spread, tracheostomy, four surgical operations annually or >10 surgeries in total. DNA was extracted from formalin-fixed paraffin-embedded tissue. HPV genotyping was performed by quantitative PCR assay identifying 15 HPV genotypes. The study included 224 patients. The majority were males (141/174 in AoRRPs and 31/50 in JoRRPs; p = 0.005). The median follow-up from initial diagnosis was 12.0 years (IQR 3.7–32.9) for JoRRPs and 4.0 years (IQR 0.8–11.7) for AoRRPs. The disease was more aggressive in juveniles than adults (p<0.001), a difference that disappeared after 10 years' observation. JoRRPs with aggressive disease were younger at onset (mean difference 4.6 years, 95%CI [2.4, 6.8], p = 0.009). HPV6 or −11 was present in all HPV-positive papillomas. HPV11 was more prevalent in aggressive disease, and HPV6 in non-aggressive disease (p<0.001). Multiple logistic regression revealed that only age at onset (OR = 0.69, 95% CI [0.53, 0.88], p = 0.003) was associated with aggressive disease in juveniles, while HPV11 (OR = 3.74, 95% CI [1.40, 9.97], p = 0.008) and observation time >10 years (OR = 13.41, 95% CI [5.46, 32.99[, p<001) were risk factors in adults. In conclusion, the only significant risk factor for developing aggressive disease in JoRRPs was age at onset, but both HPV11 and observation time >10 years were risk factors for an aggressive disease course in AoRRPs.

## Introduction

Recurrent respiratory papillomatosis (RRP) is caused by persistent infection of the respiratory epithelium by human papillomavirus (HPV): HPV6 and-11 [1]–[6]. The condition is rare [7]–[9] and characterized by recurrent growth of benign papillomas in the respiratory tract, most commonly in the larynx [10], [11]. According to the age at onset, two types of RRP are recognized; juvenile- (JoRRP) and adult-onset (AoRRP). Condylomas during pregnancy are considered the most important risk factor for acquiring JoRRP by vertical HPV transmission from mother to child [12]. In adults viral transmission may occur during oral sex [13], [14], but re-activation of a latent HPV infection acquired in childhood is another possible cause [15].

Currently there is no curable treatment for RRP and surgical procedures are required to improve voice quality and to prevent respiratory obstruction [11], [16]. The disease burden is high, and numerous hospital admissions are often necessary. The clinical course of RRP is unpredictable, frequently relapsing, and may be lifelong.

During the last two decades an increasing incidence of genital warts [17]–[20] and HPV-positive oropharyngeal carcinomas [21], [22] have been reported in the Western world. There is no surveillance of genital warts in Norway as there is in the UK and USA, but similar trends are reported in Scandinavia for both genital warts [23] and oropharyngeal carcinomas [24], [25]. It is reasonable to expect similar trends in RRP, but currently we have no evidence of such [8], [9].

We do not know why only a very few of those exposed to HPV develop RRP. Furthermore, what causes an aggressive versus an indolent clinical course is unclear, but host genetic susceptibility and genetic variability in the viral genomes have been postulated [26]. It is anticipated that the juvenile type is more aggressive than the adult type [27], [28]. In addition, several publications have found HPV11 as one of the most important risk factors for developing an aggressive disease [1], [4], [11], [29]–[32]. However many studies suffer from small sample size and the findings have not been consistently replicable [2], [26], [33], [34]. The aims of this study were primarily to describe the clinical course in our Norwegian patient cohort and secondly to explore whether gender, age at onset and HPV genotype are risk factors for developing aggressive clinical course and complications.

## Materials and Methods

### Study population

As described previously [6], patients from all regions of Norway who were treated at the the otorhinolaryngology departments at Oslo University Hospital or Lovisenberg Diaconal Hospital during 1987–2009 were recruited to the study. Patients were identified through hospital registry systems, and their records were reviewed by two laryngologists. Only patients with histopathologically-verified laryngeal papillomatosis were included. Clinical records and histological reports were reviewed. The observation time for each patient was defined as the time from the first verified biopsy of papillomatosis to the last consultation in hospital. Follow-up data were recorded to 1 January 2012.

### Clinical course and definitions

Clinical data obtained from patient records comprised gender, age at disease onset (<18 years, JoRRP; ≥18 years, AoRRP), date of first endolaryngeal procedure and biopsy, date of last follow-up, total number of endolaryngeal procedures, number of cidofovir injections, the anatomical extent of the disease at onset and the maximum extent during the observation period. Onset of the disease was defined as the first date when RRP was verified histopathologically. Complications such as tracheostomies and synecias were recorded. To provide an overall assessment of disease severity, patients were categorized as having ‘aggressive’ or ‘non-aggressive’ disease. Aggressive disease was defined as the presence of any of the following criteria: ≥10 total procedures, ≥4 procedures per year at any time, distal spread (involvement of the trachea or lung), or ever had tracheostomy. If none of these criteria were met, the patient was considered to have non-aggressive disease. This binomial classification and definition is based on data from Doyle *et al.* [28], and has been applied clinically by other researchers [1], [26], [33].

Severity of disease was also categorized by the anatomical extent of the disease according to a severity score based on a modified version of a staging assessment developed by Derkey *et al.* [35] (Table 1). The staging score for each patient was recorded at disease onset and at the maximum extent of the disease during the observation period.

**Table 1:** Staging score system.

	POINTS
**Levels involved in the larynx:**	
One point for each level	
Supraglottic	
Glottic	
Subglottic	
**Vocal folds involvment:**	
Unilateral = 1 point, Bilateral = 2 points	
**Anterior commisure:**	
No = 0 points, Yes:1 point	
**Posterior commisure**	
No = 0 point, Yes = 1 point	
**Type of papilloma**	
Single = 1 point, Multiple = 2 points	
**Involvment of trachea**	
No = 0 points, Yes = 1 point	
**Involvment of lungs**	
No = 0 points, Yes = 1 point	
**Other subsites**	
One point each site:	
Nose	
Soft palate	
Pharynx	
Other	
**TOTAL SCORE:**	
(Maximum 15 points, Minimum 3 points)	

### Histology review and selection of study samples

As described previously [6], one biopsy specimen from each patient was selected for blinded histological review by an experienced head and neck pathologist, followed by DNA extraction and HPV genotyping.

### DNA extraction & HPV genotyping

DNA was extracted from formalin-fixed paraffin-embedded tissue and HPV genotyping were performed by quantitative polymerase chain reaction (qPCR) assay method according to Lindh *et al.* [36] as previously described [6]. The DNA quality was approved in 221/224 cases. In patients with co-infections of two or more HPV genotypes, the HPV genotype with highest virus load was considered as the dominant HPV genotype. Assessment of virus load was based on the difference in concentration between genomic DNA and viral DNA. Following whole genome amplification, DNA from HPV-negative samples was sequenced using metagenomic sequencing as previously described [6].

### Statistical analysis

The patient cohort is presented by descriptive statistics for numerical and categorical data. Independent sample t-test was used for comparison of means. When normal assumption was not fulfilled, the Mann-Whitney U test was used. For identifying risk factors for aggressive disease categorical data were initially analyzed by Pearson's Χ^2^ test and significant variables was selected. Multiple logistic regression was then conducted with aggressive disease (yes/no) as dependent variable and the selected variables as independent variables (HPV6 or −11 genotype, observation time). Age at onset and gender were also included as independent variables. Statistical analyses were performed using the software package SPSS 18 (IBM Corp, Armonk, USA) with the level of significance set at p<0.05.

### Ethics statement

The study was approved by the regional Ethics Committee of South Eastern Norway Regional Authority. The Ethics Committee waived the need for informed consent for the use of clinical records, histological review and tissue samples obtained from prior diagnostics of these patients at Oslo University Hospital and Lovisenberg Diaconal Hospital. However, an information letter to each patient or the next of kin was required for use of specified data described above and so that the patients were linked to the Norwegian Cancer registry [37]. Data were anonymized and de-identified prior to analysis and processed according to the requirements of the ethical committee.

## Results

In total, clinical records and histological reports from 238 patients with RRP were reviewed. Fourteen patients were excluded from the study since paraffin blocks for HPV genotyping were not available. Statistical analyses revealed no significant differences in gender, age at diagnosis or duration of observation time in the 14 excluded patients compared to the study population. In the 224 patients included in the analysis, 50 were JoRRPs and 174 were AoRRPs. The majority of patients were male: 141 (81%) in AoRRPs and 31 (62%) in JoRRPs (p = 0.005). Median age at onset for juveniles and adults was 4.0 years (interquartile range [IQR], 2.0–6.0, maximum 17 years) and 34.0 years (IQR, 27.5–43.0, maximum 85 years), respectively. The individual observation time varied over a wide range. JoRRPs were observed at a median of 12.9 years after initial diagnosis (IQR, 3.7–32.9, maximum 62.2 years). AoRRPs had a median follow-up of 4.0 years (IQR, 0.8–11.7, maximum 45.7 years) (Table 2). The number of surgical operations per year during the observation time did not differ between AoRRP and JoRRP, but a significantly higher frequency of operations was observed in JoRRPs during the first three years after onset (Table 2). Staging scores, both at onset and at the time of maximum extent of the disease during the observation period, showed a more extensive spread in JoRRPs than AoRRPs (Table 2). Due to the wide variation in the duration of observation, patients were stratified by observation time (<10 years or ≥10 years). The observed differences in clinical course between JoRRPs and AoRRPs remained significant (data not shown). Maximum staging score in AoRRPs during the observation period was significantly higher than at onset. This was not the case in JoRRPs, where the maximum staging score during the observation period was similar to the score at onset. In AoRRPs there was a significantly lower frequency of surgical operations during the first three years compared to the total observation period, with the opposite for JoRRPs (Table 2).

**Table 2:** Patient characteristics, n = 224.

	JoRRP n = 50	AoRRP n = 174	P value
	Median (IQR)	Median (IQR)	
Observation time (years)	12.9 (3.7, 32.9)	4.0 (0.8, 11.7)	
Number of surgical procedures per year^a^	1.2 (0.5, 3.4)^b^	1.2 (0.6, 2.6)^c^	
Number of surgical procedures per year during the first three years	2.3 (1.0, 3.8)	1.0 (0.5, 1.76)	p<0.001
Staging score at onset	7.0 (6.0, 7.0)	5.0 (4.0, 6.0)^d^	p<0.001
Maximum staging score	7.0 (6.0, 7.5)	6.0 (5.0, 6.0)	p<0.001

a During the observation period.

b The operation frequency in JoRRP during the observation time was lower than during the first three years (p = 0.005).

c The operation frequency in AoRRP during the observation time was higher than during the first three years (p = 0.031).

d The difference in staging score at onset and maximal score during observation time in AoRRP was significant (p = 0.050).

AoRRP, adult recurrent respiratory papillomatosis; IQR, interquartile range; JoRRP, juvenile recurrent respiratory papillomatosis.

Ten of the included patients were treated with Cidofovir injections. The treatment was given as 5–7.5 mg/ml submucosal injections in the larynx preoperatively in the study period from 2006–2009. One JoRRP patient categorized as aggressive was given Cidofovir as an adult 41 years after disease onset. The nine others were AoRRPs; 6 with aggressive disease and 3 with non-aggressive disease given in average 4.2 years after disease onset. In average 3.2 injections were given in each patient, varying from two to maximum seven injections. The patients had an average observation time of 2.5 years after the last injection. All of them had recurrences after the treatment and showed no substantially change in the clinical course.

The distribution of glottic involvement and complications were significantly higher in JoRRPs than AoRRPs (Table 3). After stratification for observation time, the differences were still significant for the first ten years except for distal involvement, which was more prevalent in juveniles regardless of disease duration (data not shown). When disease severity was categorized as either aggressive or non-aggressive, an aggressive disease course was significantly more frequent in juveniles than adults: 37/50 (74%) of JoRRPs versus 51/174 (29.3%) of AoRRPs (p<0.001). Over time (≥10 years), these differences disappeared. Males and females showed similar frequencies of aggressive disease. In juveniles the mean age at onset was significantly lower in patients with aggressive disease compared to non-aggressive (mean age difference 4.6 years, 95% CI 2.4, 6.8, p = 0.009), but no such difference in adults.

**Table 3:** Distribution of glottic involvement and complications of the disease stratified for juvenile and adults during observation time, n (% within adult/juvenile group), n = 224.

Glottic involvement/complications	Juvenile-onset RRP	Adult-onset RRP	P value
	n = 50 (%)	n = 174 (%)	
Involvement of ant.commisure^a^	37 (86.9)	86 (58.9)	p = 0.001
Bilateral involvement of the vocal folds^b^	40 (93.0)	103 (71.5)	p = 0.004
Distal involvement^c^	5 (10.0)	5 (2.9)	p = 0.032
Tracheostomies	5 (10.0)	2 (1.1)	p = 0.002
Synechias	22 (44.0)	8 (16.1)	p<0.001

a N = 189, 35 patients had missing recordings.

b N = 187, 37 patients had missing recordings.

c Including involvement of trachea or lungs.

RRP, recurrent respiratory papillomatosis.

HPV was detected in 207 (93.7%) of the 221 patients for whom DNA quality was approved. Of these 207 patients, 133 were positive for HPV6, 40 for HPV11 and 15 for both. Co-infection with one or two high-risk HPV types, as well as HPV6 or HPV11, was present in 19 patients. In 14/221 patients, HPV was not detectable by qPCR. Metagenomic sequencing of these 14 HPV-negative patients found HPV8 in one case; the remaining 13 cases were HPV negative. In patients with multiple HPV infection, either HPV11 or HPV6 showed highest viral load. Patients were therefore analyzed according to their dominant HPV genotype. We found a higher rate of HPV11 in juveniles than adults, and the converse for HPV6, but these differences were non-significant (p = 0.054) (Table 4) There was a significantly higher rate of HPV11 among the patients with aggressive disease and a higher rate of HPV6 among those with a non-aggressive course (p<0.001) (Table 5). When stratifying for juveniles and adults, there was a significantly higher rate of HPV11 among the aggressive versus non-aggressive in adults (p = 0.003), but no significant difference among juveniles (p = 0.358).

**Table 4:** Distribution of HPV6 and HPV11 (n = 207^a^).

	HPV11	HPV6
	n (%)	n (%)
Juvenile-onset RRP	16 (33.3)	32 (20.0)
Adult-onset RRP	32 (66.7)^b^	127 (80.0)
Total	48 (100.0)	159 (100.0)

a Excluding one HPV8-positive patient.

b p = 0.057 for the prevalence of HPV11 in juveniles versus adults.

**Table 5:** Distribution of HPV6 and HPV11 in patients (including AoRRPs and JoRRPs) with an aggressive or non-aggressive clinical course (n = 207)^a^.

	HPV11	HPV6	Total
	n (%)	n (%)	
Aggressive	29 (60.4)	52 (32.7)	81
Non-aggressive	19 (39.6)	107 (67.3)	126
Total	48 (100.0)	159 (100.0)	207

a Excluding one HPV8-positive patient.

The difference in HPV profile between the two subgroups was significant (p<0.001).

A multiple logistic regression model was constructed to evaluate which risk factors were most closely related to risk of aggressive disease. Independent variables included in the model were gender, age at onset, genotypes HPV6 and HPV11, and observation time. Analyses were performed separately for juveniles and adults. Only age at onset was a significant predictor of aggressive disease among juveniles. In adults, HPV11 and observation time ≥10 years were both significant risk factors. Gender was non-significant in both juveniles and adults (Table 6).

**Table 6:** Risk factors for an aggressive clinical course in juvenile and adult onset RRP.

Variable	Unadjusted OR (95% CI)	P value	Adjusted (95% CI)	P value
**Juveniles**				
**Age at onset**	**0.70 (0.55, 0.89)**	**0.004**	**0.69 (0.53, 0.88)**	**0.003**
Male	1			
Female	1.53 (0.39, 5.91)	0.534	3.49 (0.40, 30.08)	0.256
HPV 6	1			
HPV 11	2.11 (0.55, 8.22)	0.279	1.52 (0.25, 9.42)	0.651
Observation time	1			
<10 years				
Observation time	3.54 (0.89, 14.06)	0.073	3.91 (0.70, 21.78)	0.119
≥10 years				
**Adults**				
Age at onset	0.98 (0.96, 1.01)	0.200	1.01 (0.98, 1.05)	0.426
Male	1			
Female	0.88 (0.38, 2.06)	0.780	0.74 (0.25, 2.17)	0.586
HPV 6	1			
**HPV 11**	1.23 (0.84, 1.81)	0.283	**3.74 (1.40, 9.97)**	**0.008**
Observation time	1			
<10 years				
**Observation time**	**11.21 (5.19, 24.22)**	**<0.001**	**13.41 (5.46, 32.99)**	**<0.001**
**≥10 years**				

Significant risk factors are highlighted in bold.

CI, confidence interval; OR, odds ratio; RRP, recurrent respiratory papillomatosis.

## Discussion

To our knowledge, this is the first study to compare the clinical course and HPV genotype status of RRP in both juveniles and adults. During the last 15 years, large multicenter studies [8], [11], [38] and several smaller cohort studies and case series [1], [2], [26]–[29], [31], [33], [34], [39] in juveniles have been conducted, most of which have included HPV genotyping. Studies comprising only AoRRP or both age groups are scarce, have small sample sizes, and many carried out during the 1980s and early 1990s [28], [32], [40]–[45]. The few trials in which HPV status has been examined were generally undertaken before PCR was widely available and used less sensitive HPV detection methods such as Southern Blot and Hybridization [43]–[45]. Exceptions are the publications by Pou *et al.* [32] and Aaltonen [42], which include HPV genotyping by PCR. Two studies included both age groups, but without comparing the two groups in terms of HPV genotype or clinical course [3], [13].

As reported previously [28], our study confirmed that JoRRPs are more severely affected by frequent surgery, complications and distal spread of the disease compared with AoRRPs. Additionally, we found that young age at onset among juveniles was associated with aggressive disease, consistent with previous studies [26], [39], [46]. Age at onset in adults was not associated with an aggressive clinical course, in contrast to the findings of Aaltonen *et al.* [41] who found young age at onset to be a significant risk factor for disease severity. Their outcome measure was the total number of surgical operations, without correction for widely varying observation time. We used the number of operations per year as one outcome (Table 2), but also included four possible criteria for aggressive disease and stratified the outcome results by observation time with a cut-off point of ten years.

Furthermore, our study shows that when the observation time exceeds ten years, the differences in aggressiveness between juveniles and adults disappear, though not for distal spread, which is more pronounced in juveniles regardless of disease duration. Moreover, we observed that in adults the maximum extent of the disease may progress after onset, while in juveniles it does not increase. This is in accordance with the operation frequency in adults (Table 2), which is significantly higher during the entire observation time than in the first three years. This may be explained by a different approach to treatment: juveniles have surgeries scheduled every 4–5 weeks until the papillomas regress. In adults, the approach is more pragmatic, with surgery scheduled only when the patient has complained of hoarseness. Many adult patients are accustomed to being hoarse and therefore may wait an unnecessarily long time before seeking medical advice.

Several publications have shown that HPV11 is one of the most important risk factors for developing an aggressive clinical course [1], [4], [11], [29]–[32], but this finding has not been consistently replicated [2], [26], [33], [34]. Additionally, HPV11 appears more frequently in children than in adults [32], [42], such that is difficult to conclude whether the aggressiveness in juveniles is driven by HPV11 *per se* or whether HPV11 represents a confounder to an unknown susceptibility in children, e.g. an immature immunological response. Regarding the distribution of HPV6 and HPV11, there was a trend of a higher proportion of HPV11 in juveniles and HPV6 in adults. These differences did not reach significance level (p = 0.054), which may be due to the small sample size. Our figures showed that HPV11 occurred more frequently with aggressive versus non-aggressive disease (p<0.001). However, when stratified for juveniles and adults, the difference in the distribution of HPV6 or HPV11 was significant only in adults (p = 0.004). This is supported by the logistic regression model, which showed age at onset to be the only significant risk factor for aggressive disease in juveniles, whereas in adults both HPV11 and observation time ≥10 years were significant.

Another aspect of the difference in the HPV genotype distribution between JoRRPs and AoRRPs is that it is consistent with the hypothesis that there are different modes of viral transmission in adults versus juveniles. If HPV virus acquired in childhood is reactivated in adulthood, we would expect to find the same HPV profile in AoRRPs and JoRRPs. Hence, a different distribution of HPV6 and HPV11 between AoRRPs and JoRRPs supports the concept of vertical HPV transmission from mother to child in JoRRPs [12] while AoRRPs are infected in adulthood, possibly during oral sex [13], [14].

As far as we know, there is no standardized staging or assessment system for RRP. In contrast to the staging system proposed by Derkay *et al.* [35], where clinical course is included, our assessment system does not implicate criteria other than the anatomical extent of the disease. Our assessment is also less detailed, resulting in a simplified scoring. We believe that a detailed staging system such as that of Derkay and colleagues is useful for the surgeon to evaluate whether the disease has progressed when scheduling surgery. In research, subtle changes between each surgical procedure will probably be difficult to detect, as reflected in the study of Altonen *et al.* [41] in which neither symptoms nor size and number of lesions was predictive of the clinical disease course.

The limitation of our study lies in the retrospective sampling of data from patient records. We assume correct and accurate data, but we cannot rule out information bias. Moreover, the staging score was only registered at the time of disease onset and at the time of maximum extent of the disease. We were able to register the operation frequency, but we did not have complete data to register the date and staging score for each surgical intervention, which would have given an even more detailed picture of the clinical course.

As in most follow up studies it is a common problem that patients are given treatment that may influence the endpoints, such as Cidofovir treatment in this study. Additionally, risk factors that are studied may also potentially influence whether the patients are given treatment or not. This problem can not be resolved by statistical methods or excluding patients and this is one of the reasons why follow-up studies are less reliable than randomized studies.

The strength of our method is that this was a relatively large cohort of both AoRRPs and JoRRPs, with patients enrolled from only two hospitals, thus reducing the likelihood of information bias.

## Conclusions

The clinical course of RRP in juveniles is more severe than in adults. There is a higher rate of HPV11 genotype compared to HPV6 among patients with an aggressive disease course compared to non-aggressive disease. In AoRRPs, the risk factors related to aggressive disease were HPV11 genotype and observation time exceeding 10 years, while in JoRRPs only age at onset was of significance.
